# Optical properties of silicon nanowire arrays formed by metal-assisted chemical etching: evidences for light localization effect

**DOI:** 10.1186/1556-276X-7-524

**Published:** 2012-09-25

**Authors:** Liubov A Osminkina, Kirill A Gonchar, Vladimir S Marshov, Konstantin V Bunkov, Dmitry V Petrov, Leonid A Golovan, Florian Talkenberg, Vladimir A Sivakov, Victor Yu Timoshenko

**Affiliations:** 1 Physics Department, Lomonosov Moscow State University, Leninskie Gory 1, Moscow, 119991, Russia; 2 Skobeltsyn Institute of Nuclear Physics (MSU SINP), Lomonosov Moscow State University, Leninskie Gory 1(2), Moscow, 119234, Russia; 3 Institute of Photonic Technology, Albert-Einstein Street 9, Jena, 07745, Germany

**Keywords:** Silicon nanowires, Black silicon, Raman scattering, Light localization

## Abstract

We study the structure and optical properties of arrays of silicon nanowires (SiNWs) with a mean diameter of approximately 100 nm and length of about 1–25 μm formed on crystalline silicon (c-Si) substrates by using metal-assisted chemical etching in hydrofluoric acid solutions. In the middle infrared spectral region, the reflectance and transmittance of the formed SiNW arrays can be described in the framework of an effective medium with the effective refractive index of about 1.3 (porosity, approximately 75%), while a strong light scattering for wavelength of 0.3 ÷ 1 μm results in a decrease of the total reflectance of 1%-5%, which cannot be described in the effective medium approximation. The Raman scattering intensity under excitation at approximately 1 μm increases strongly in the sample with SiNWs in comparison with that in c-Si substrate. This effect is related to an increase of the light-matter interaction time due to the strong scattering of the excitation light in SiNW array. The prepared SiNWs are discussed as a kind of ‘black silicon’, which can be formed in a large scale and can be used for photonic applications as well as in molecular sensing.

## Background

Currently, silicon nanowires (SiNWs) are of great interest due to their potential applications in various fields suchlike microelectronics, optoelectronics, photonics, photovoltaics, biologic, and chemical sensors [1-9]. In particular, SiNWs exhibit a strong optical absorption and rather low reflectance in the visible spectral range [4,6] as well as in room temperature photoluminescence (PL) [10,11]. There are several ways to form SiNWs, and the first method is based on vapor–liquid-solid or bottom-up growth catalyzed by noble metal (mostly gold, Au), as firstly proposed by Wagner and Ellis in 1964 [12]. Some years ago, it was demonstrated that SiNWs can be formed by top-down approaches suchlike a reactive ion etching, electrochemical etching, and metal-assisted chemical etching (MACE) [13-19]. Since both the electronic and optical properties of nanostructures are dependent on the method of their preparation, it is of great importance to control the growth and structure of SiNWs.

In the present paper, we study the morphology, microstructure, and optical properties of SiNWs obtained by MACE (assisted by silver nanoparticles) of crystalline silicon (c-Si) in hydrofluoric acid solution. It is shown that the formed SiNWs are characterized by the optical reflection and transmission, which are strongly modified in comparison with those for c-Si substrate. In addition, SiNWs exhibit efficient Raman scattering, and this effect can be interpreted in terms of the strong Anderson-like localization of the excitation light in the SiNW array.

## Methods

**Figure 1:**
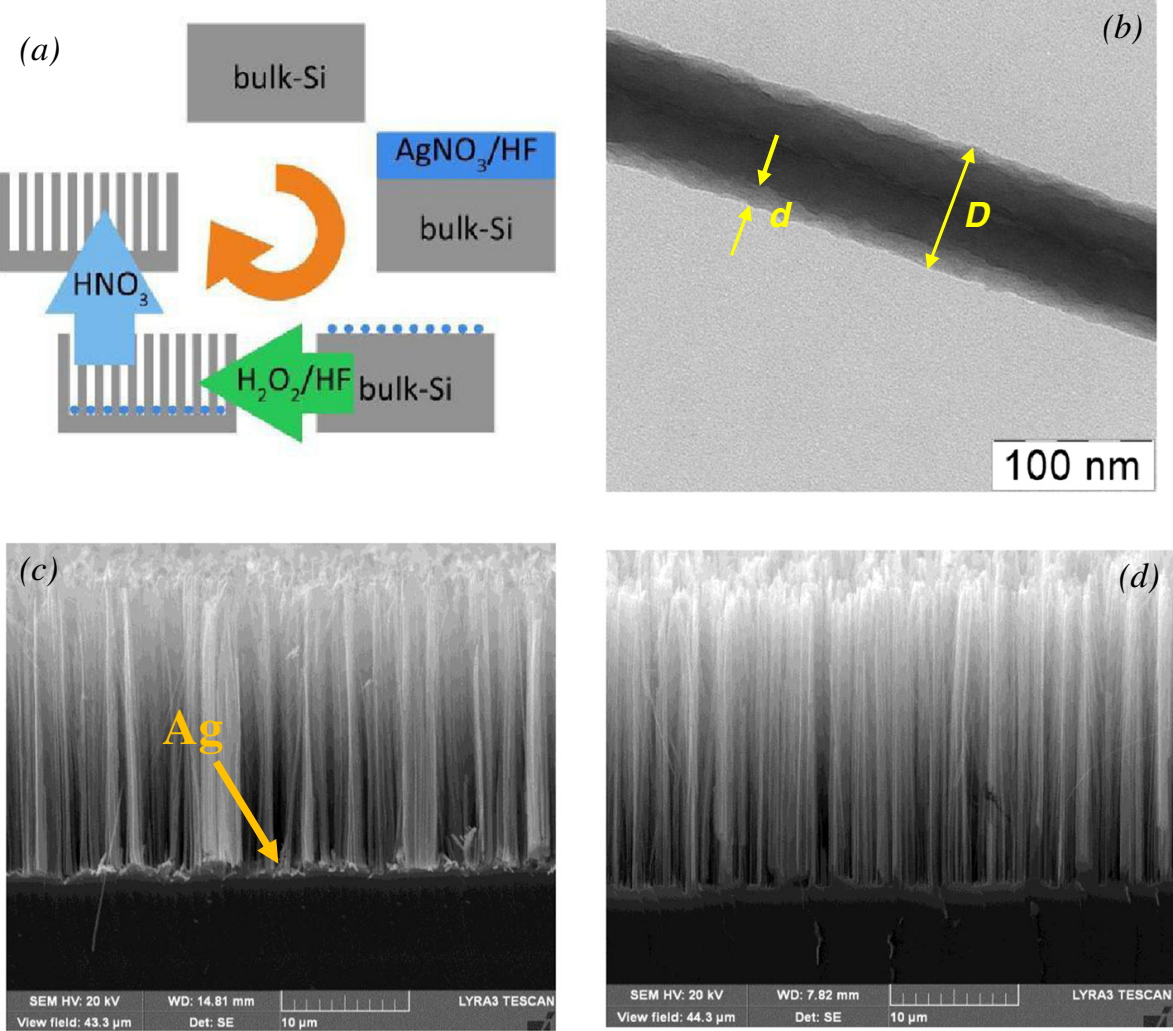
Typical TEM and SEM micrographs of SiNWs: (a) a schematic representation of the MACE procedure; (b) TEM image of an individual SiNW, where thicknesses of the whole SiNW and its nanostructured side are marked by yellow symbols D and d, respectively; and (c,d) SEM micrographs of the SiNW arrays with and without Ag nanoparticles (marked by yellow arrow).

SiNWs were prepared by MACE of p-type double-side polished (100)-oriented c-Si wafers with specific resistivity of 1–10 Ω cm. Prior the MACE procedure, the c-Si substrates were rinsed in 49% HF solution for 1 min to remove native oxide. Then in the first step of MACE, polycrystalline silver nanoparticles of different morphology were deposited on the substrates by immersing them in aqueous solution of 0.02 M of silver nitrate (AgNO3) and 5 M of HF in the volume ratio of 1:1 for 30 s (see for details [19]). In the second step, the c-Si substrates covered with Ag nanoparticles were immersed in the solution containing 5 M of HF and 30% H_2_O_2_ in the volume ratio of 10:1 in a Teflon vessel for time varied from 1 to 30 min, which resulted in the length of SiNWs from 2 to 60 μm, correspondingly. All etching steps were performed at ambient temperature. Finally, the samples were rinsed several times in deionized water and dried at room temperature. A part of the formed SiNW arrays was additionally immersed in concentrated (65%) nitric acid (HNO_3_) for 15 min to remove residual Ag nanoparticles from the SiNWs. Figure 1a shows the step-by-step formation of SiNWs.

The structural properties of SiNWs were investigated by using a scanning electron microscope (SEM) of Lira Tescan (Tescan USA, PA, USA) and a transmission electron microscope (TEM) of LEO912 AB Omega (Carl Zeiss SMT AG Oberckochen, Germany). The optical reflectance and transmittance spectra in the region of 500–10,000 cm^−1^ were studied with a Fourier-transform infrared (FTIR) spectrometer of Bruker IFS 66v/S (Bruker Corporation, Billerica, MA, USA). The spectra were measured at normal incidence. The infrared (IR) PL and Raman spectra under excitation with a CW Nd:YAG laser at 1.064 μm (excitation intensity approximately 100 mW; spot size approximately 2 mm) were measured in a back scattering geometry with the same spectrometer equipped with an FRA-106 unit. The total reflectance spectra in the region from 300 to 2,000 nm were measured with Perkin Elmer Lambda 950 spectrometer (PerkinElmer Inc., Waltham, MA, USA) equipped with an integrating sphere. All measurements were carried out at room temperature in air.

## Results and discussion

Figure 1b shows a TEM image of individual SiNW. The diameter, *D*, of SiNW is about 100 nm and *D* is nearly constant through the whole SiNW length, which was varied from 2 to 60 μm in the prepared samples. The outer surface of SINW is rough in nanoscale [10] and the nanostructure surface layer, *d*, is visible as less dark regions in the TEM image of SiNW sides.

Figure 1b,c shows typical large-scale cross-sectional SEM images of a sample with SiNWs. One can see that SiNWs look like quasi-ordered arrays with preferential orientation along the [100] crystallographic direction. The length of SiNWs, *L*, was nearly proportional to the etching time in agreement with results of [18].

Since the growth of SiNWs is controlled by the redox process initiated by silver (Ag) nanoparticles, they are observed at the interface SiNWs/c-Si for as-prepared samples (Figure 1c). The Ag nanoparticles could be easily removed from the interface by etching the samples in concentrated HNO_3_ (see Figure 1d). It is important to note that the etching in HNO_3_ did not lead to remarkable changes of the structure properties of SiNWs monitored by means of TEM and SEM.

**Figure 2:**
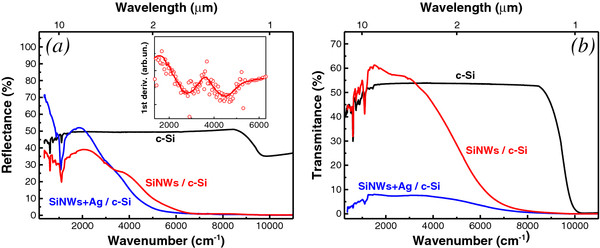
**Reflectance and transmittance spectra: (a) the reflectance of double-side polished c-Si substrate (black curve) and a sample of SiNWs (*L* = 2.6 μm), with and without Ag nanoparticles (blue and red curves, respectively), and the first derivative of the reflectance of the sample of SiNWs without Ag nanoparticles in the inset; (b) the transmittance of double side polished c-Si substrate (black curve) and a sample of SiNWs (*L* = 2.6 μm) with and without Ag nanoparticles (blue and red curves, respectively).**

The FTIR reflectance spectrum of the bare substrate (Figure 2a) corresponds to the well-known behavior for the double side polished c-Si wafer. The reflection coefficient was about 30%-40% in the region of the interband absorption (wavelength, λ < 1 μm). In the transparency region (λ > 1 μm), the reflection value increases because of the both sides contribution in c-Si wafer.

The reflection coefficient (Figure 2a) and transmittance (Figure 2b) of SiNWs are rather high in the spectral region of wave numbers below 5,000 cm^−1^ (λ > 2 μm). Since in this spectral region *D* < < λ the SiNW array can be considered as an effective optical medium with the effective refractive index *n*_eff_. The latter can be estimated from the interference oscillations in the first derivative of the reflection coefficient (see inset in Figure 2a) and it is accounted to be 1.3 ± 0.1 in the spectral range of 2,000-6,000 cm^−1^. According to the effective medium approximation (Bruggeman model, see for example review [20]), the value of *n*_eff=_1.3 corresponds to the porosity of SiNW array to be about *P* = 75%. This value seems to be formed by both the spatial density of SiNW array and the local porosity of SiNW sides. Note that the reflectance is higher and the transmittance is lower for the samples with Ag nanoparticles (blue curves in Figure 2). This effect is obviously explained by the contribution of the metal fraction to the effective dielectric function. Both the reflection coefficient (Figure 2a) and the transmittance (Figure 2b) of SiNWs decrease in the high frequency range (λ < 2 μm) due to the light scattering.

**Figure 3:**
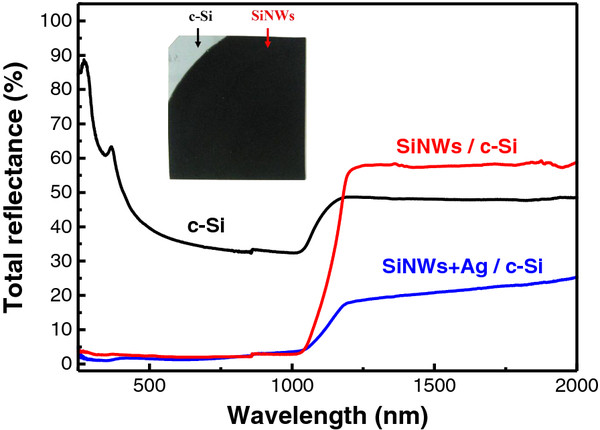
**Total reflectance spectra of the double side polished c-Si substrate and a sample of SiNWs.** Total reflectance spectra of the double side polished c-Si substrate (black curve) and a sample of SiNWs (*L* = 2.6 μm) with and without Ag nanoparticles (blue and red curves, respectively). Inset shows a digital photo of the corresponding sample.

Figure 3 shows the total reflectance spectra of SiNWs and c-Si substrate. The total reflectance of c-Si is near 30%-40% for the visible spectrum region, opposite to the samples with SiNWs, which exhibit a strong decrease of the total reflectance to 1%-5%. So, in the visible and near IR spectral regions the samples with SiNWs are looking similar to “black silicon” (see inset in Figure 3). This property was observed for all samples with SiNWs (*L* = 2–60 μm), and it can be explained by the strong light scattering, which results in partial localization (trapping) of the excitation light in SiNW array.

**Figure 4:**
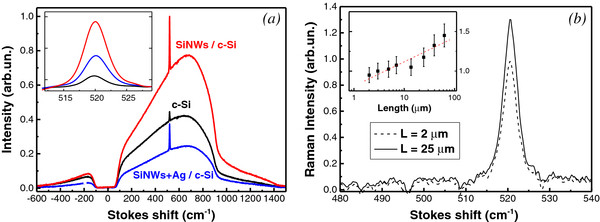
**Infrared photoluminescence and Raman spectra: (a) the PL and Raman spectra of double side polished c-Si substrate (black curve) and a sample of SiNWs (*L* = 2.6 μm) with and without Ag nanoparticles (blue and red curves, respectively), and the inset shows the Raman peak after subtraction of the PL background; (b) Raman spectra the samples of SiNWs with two different thicknesses, and the inset shows dependence of the Raman intensity on the length of SiNWs (dashed red curve corresponds to a fit by the logarithmic function).**

The effect of light localization can be studied in more details by analyzing the Raman spectra of SiNWs. Figure 4a shows the typical spectra of the PL and Raman scattering of two samples with SiNWs (with and without Ag nanoparticles) and c-Si substrate for comparison. The spectra are represented versus the Stokes shift in respect to the excitation light. Since the excitation was done by the laser irradiation at 1.064 μm, the corresponding region ±100 cm^−1^ near the excitation line was rejected by a notch filter. The Raman scattering is observed as a sharp peak at 520 cm^−1^, which is superimposed on the broad PL band related of the radiative recombination of photo-excited charge carrier near the indirect band gap of c-Si (approximately 1.12 eV [21]). The PL band position and shape for the samples with SiNWs are similar to those for the c-Si substrate. This fact is well understood by taking into account that the SiNW’s diameter, *D*, at approximately 100 nm and it is far from the quantum confinement regime [22]. The bimolecular mechanism of the interband radiative recombination in SiNWs with similar *D* has been recently demonstrated in our previous work [11].

The Raman scattering intensity increases strongly for the samples with SiNWs in comparison with the corresponding value of c-Si substrate (see inset in Figure 4a). The enhancement of the Raman scattering is several times larger for the samples without Ag nanoparticles. This fact can be explained by taking into account the additional optical losses induced by Ag nanoparticles, while the well-known effect of the local field enhancement near metallic particles seems to be negligible in the investigated structures.

Figure 4b shows that the Raman scattering intensity of SiNWs is not proportional to the length of SiNW. The corresponding dependence is well approximated by a logarithmic function as it is shown in the inset of Figure 4b. This logarithmic dependence indicates a diffusive origin of propagation of the excitation radiation and/or detected light. It is worth to notice that the Raman scattering from the samples with SiNWs was completely depolarized, while the signal of the substrate demonstrated the characteristic polarization dependence, which was inherent for the (100)-oriented c-Si wafer [11].

In order to get a quantitative measure of the Raman scattering enhancement in SiNWs, let us to consider the Raman enhancement per unit volume (RE) similarly to the measurement proposed in [23]:

(1) $$\text{RE}=\frac{\left[I_{\text{SiNW}}/V_{\text{SiNW}}\right]}{\left[I_{\text{bulk}}/V_{\text{bulk}}\right]}\text{,}$$

Where *I*_SiNW_ and *I*_bulk_ are the measured Raman intensities for the samples with SiNWs and for the c-Si substrate, respectively; *V*_SiNW_ and *V*_bulk_ are the corresponding SiNWs and the c-Si substrate volumes probed by the excitation light. Because the Stokes component of the Raman scattering in c-Si excited at 1.06 μm is weak-absorbed (absorption coefficient at approximately 2 cm^−1^[21]), the value of *V*_bulk_ is about the thickness of c-Si substrate, which was equal to *L*_0_ = 400 μm, multiplied by the excitation spot area (*S ≈* 4 mm^2^). The excited volume of SiNWs is approximately proportional to the length of SiNWs corrected to the porosity, i.e., $V_{\text{SiNW}}\approx \frac{L\;S}{\left(1-P\right)}$.

According to Equation 1, RE is approximately 10^3^ for the sample of SiNWs with *L* = 2 μm. The value of *RE* decreases for the samples with larger *L*. This fact indicates that the main contribution to the Raman scattering enhancement is induced on the thin (*L* < 2 μm) top layer of the SiNW arrays.

The enhancement of the Raman scattering in SiNWs can be interpreted as increasing the excitation intensity of SiNWs because of the partial light localization in inhomogeneous optical medium [11]. Such kind of light localization was observed in different porous semiconductors as GaP, TiO_2_, and Si (see for example a review in [20] and references therein); and it is analogous for the Anderson localization for electrons in amorphous semiconductors [23].

Another explanation of the enhanced Raman scattering in SiNW array is based on consideration of a resonant enhancement of the incident electromagnetic radiation by individual SiNWs as demonstrated in [24]. While both explanations are possible, the concept of light localization allows us to understand better the observed dependence of the Raman scattering intensity on *L* (see Figure 4b).

In the framework of the light localization concept [11,25], the enhancement of the Raman scattering implies a strong increase of the effective interaction time of exciting photon with the samples, which can be estimated by the following relation:

(2) $$\tau_{\mathrm{SiNW}}\approx RE\cdot \;\tau_{\mathrm{bulk}}\text{,}$$

where $\tau_{\mathrm{bulk}}=\frac{2L_{0}n_{\mathrm{bulk}}}{c}$ is the interaction time of photon with the c-Si substrate (*c* is the velocity of light in vacuum; *n*_bulk_ is the refractive index of c-Si). Since for the employed substrate $\tau_{\mathrm{bulk}}\approx 10^{-11}$ s we can estimate the lower limit of *τ*_SiNW_ at approximately 10^-8^ s.

The strong shortening of light-matter interaction time in SiNW array is obviously interesting for various photonic applications as nonlinear optical conversion of the light frequency and for the duration of short laser pulses [20]. Furthermore, the extremely low total reflectance of SiNW array in the ultraviolet (UV)-visible and near IR spectral ranges can be used to increase the absorbance of solar cells and other photovoltaic devices. Indeed, the application of SiNWs for new type of solar cell was already demonstrated [6].

Another field of application of the observed effect of light localization is the sensing of molecules incorporated or/and absorbed in SiNW array. The detection of enhanced molecular response at characteristic vibration frequencies can be realized by means of the IR reflectance/transmittance and Raman spectroscopy. The possibility of the latter was recently demonstrated for grooved Si structure with partial light localization [26].

## Conclusions

The obtained experimental data on the structure and optical properties of silicon nanowire arrays prepared by metal-assisted chemical etching of c-Si in hydrofluoric acid solutions demonstrate new possibilities for tailoring the physical properties of Si-based nanostructures. The reflectance and transmittance of SiNWs depend on the presence of Ag nanoparticles, which result in the optical losses in the samples. In the middle IR spectral range, the formed arrays of SiNWs act as the effective medium with the low refractive index, which can be used as anti-reflective coating for photonic devices. The formed SiNW arrays demonstrate a strong decrease of the total reflectance near 1%-4% in the spectral region of 300 ÷ 1,000 nm and they can be used as a kind of ‘black silicon’, which can be formed by the simple chemical method in a large scale. The observed low reflectance correlates with the strong increase of the Raman scattering efficiency in SiNWs in comparison with that for c-Si substrate. The enhancement of the Raman scattering efficiency is interpreted as an evidence of the light localization because of the multiple scattering in SiNW array. The light localization effect in SiNWs can be used in various photonic applications as well as in molecular sensorics.

## Competing interests

The authors declare that they have no competing interests.

## Authors’ contributions

LAO performed the SiNWs fabrication, optical measurements, and data analysis. KAG and LAG performed structural measurements and data analysis of the reflectance spectra of the samples. KVB measured the Raman spectra. VSM performed the SiNWs fabrication and calculate the growth rate of SiNWs. DVP performed the SEM measurements. FT and VAS contributed in the development of the preparation method and analysis of the results. VYT performed the general data analysis and discussion of the obtained data. All authors read and approved the final manuscript.
